# First Report of Caseous Lymphadenitis by *Corynebacterium pseudotubercolosis* and Pulmonary Verminosis in a Roe Deer (*Capreolus capreolus* Linnaeus, 1758) in Italy

**DOI:** 10.3390/ani14040566

**Published:** 2024-02-08

**Authors:** Alessandra Di Donato, Lorenzo Gambi, Valentina Ravaioli, Simona Perulli, Letizia Cirasella, Rachele Rossini, Andrea Luppi, Giovanni Tosi, Laura Fiorentini

**Affiliations:** 1 Istituto Zooprofilattico Sperimentale Della Lombardia e dell’Emilia Romagna (IZSLER), 47122 Forlì, Italy; alessandra.didonato@izsler.it (A.D.D.); lorenzo.gambi27@gmail.com (L.G.); valentina.ravaioli@izsler.it (V.R.); simona.perulli@izsler.it (S.P.); rachele.rossini89@gmail.com (R.R.); giovanni.tosi@izsler.it (G.T.); laura.fiorentini@izsler.it (L.F.); 2 Istituto Zooprofilattico Sperimentale Della Lombardia e dell’Emilia Romagna (IZSLER), 43126 Parma, Italy; andrea.luppi@izsler.it

**Keywords:** caseous lymphadenitis, *Corynebacterium pseudotubercolosis*, roe deer, *Capreolus capreolus*, abscesses

## Abstract

**Simple Summary:**

This case report describes the first case of caseous lymphadenitis in a roe deer (*Capreolus capreolus*, Linnaeus 1758) found dead in Italy, attributable to *Corynebacterium pseudotuberculosis* isolated from abscessual and non-abscessual lesions of the lungs and lymph nodes. In addition, histological examinations revealed a severe parasitological infestation by bronchopulmonary larvae at various stages of development. These findings raise the possibility of the reservoir-like role of cervids for *C. pseudotubercolosis* and the potential implications related as much to wildlife conservation as to pathologies at the domestic/wildlife interface.

**Abstract:**

Caseous lymphadenitis is a chronic debilitating disease typical of small ruminants, but it is also noted in several other domestic and wild species. In this report, we present the first documented case in Italy of pseudotuberculosis in a roe deer (*Capreolus capreolus*, Linnaeus 1758) found dead in the mountains of Forlì-Cesena province, Emilia Romagna region. The carcass underwent necropsy according to standard protocols, revealing generalized lymphadenopathy and severe apostematous pneumonia with multifocal and encapsulated abscesses. *Corynebacterium pseudotuberculosis* was isolated from the lung parenchyma, lymph nodes and abscesses. Additionally, severe parasitic bronchopneumonia of the caudal lobes and gastrointestinal strongyle infestation were detected. To our knowledge, this is the first documented case of CLA referable to *C. pseudotubercolosis* in a roe deer in Italy.

## 1. Introduction

Caseous lymphadenitis (CLA) is a common bacterial disease in sheep and goats worldwide, caused by *Corynebacterium pseudotuberculosis*. It is a chronic, contagious, suppurative disease that may eventually be fatal [1,2]. The etiological agent is a Gram-positive bacteria belonging to the Actinomycetia class like other suppurative bacteria such as *Rhodococcus* spp. and *Mycobacterium* spp. [3]. Another frequently reported member of the genus Corynebacterium is *C. ulcerans*, which causes localized to systemic abscesses [4,5]. *C. pseudotuberculosis* can enter the body either through skin, mucous membranes, inhalation or ingestion, reaching the regional lymph nodes of the entry route [6,7]. Here, *C. pseudotuberculosis* generates caseous abscesses and infects other organs and lymph nodes via lymphatic or hematogenous routes. Abscesses can sometimes display an onion-ring-layered appearance at carving. A distinct clinical sign is the enlargement of external lymph nodes, supported by chronic weight loss [8]. Other clinical signs (e.g., coughing and respiratory symptoms) are related to the organs and systems affected. The diagnosis of symptomatic cases is based on the isolation of *C. pseudotuberculosis* from enlarged lymph nodes. Only a few commercial vaccines are available, which do not provide total protection and may display some side effects [9]. Thus, a CLA-free flock would be the best prevention measure, followed by the testing of any new animal introduced. Eradication is difficult as *C. pseudotuberculosis* can survive for months in the environment [1]. CLA has a great relevance for sheep and goat farmers as it causes great economic losses worldwide, due to the decreased reproductive efficiencies of affected animals, reductions in milk and wool yields and the condemnation of both carcasses and skins in slaughterhouses [6,10]. *C. pseudotuberculosis* is a zoonotic agent, despite being rarely encountered in humans and being described mainly after occupational exposure or contact with animals, with symptoms ranging from local lymph node abscesses to pneumonia [6,11,12,13].

*C. pseudotuberculosis* infections have been reported in several other species, both domestic and wildlife [14,15,16,17,18]. Nevertheless, *C. pseudotuberculosis* in suids has rarely been described, (e.g., an infection of pigs from two different farms in Spain) [19], as most infections in wild boars and pigs are caused by *C. ulcerans* [4,20,21], sometimes recently classified as novel *C. sylvaticum* [22,23,24]. Some evidence of CLA or *C. pseudotuberculosis* infections has been reported in other wildlife species, mainly ruminants: cervids, wild caprinae species and antelopes [25]. The majority of research has focused on wild caprinae, with numerous samples being analyzed [26,27,28]. On the other hand, the limited number of cervid studies counted few animals, describing both forms of CLA attributable to *C. pseudotuberculosis* [29,30,31,32,33] and extremely similar *C. ulcerans* infections [5]. Only one CLA case in roe deer by *C. pseudotuberculosis* has been published, observed during a fifty-year surveillance in Switzerland [34].

*Mannheimia granulomatis* is a Gram-negative small rod that is reported occasionally as a cause of granulomatous or abscessual forms in various body sites, such as the tongue, neck and respiratory tract in cattle, cervids and hares [35,36,37,38].

In Italy, there are three species of wild cervids: red deer (*Cervus elaphus*, Linnaeus 1758), fallow deer (*Dama dama*, Linnaeus 1758) and roe deer (*Capreolus capreolus*, Linnaeus 1758). The latter is the most abundant and widespread, with a distribution area that extends for about 110,000 km^2^, nearly a third of the entire country’s territory. The total roe deer population has been increasing in Italy since 1960, and the same tendency has been observed in the Emilia Romagna region. Roe deer is a significant game species in Italy and Europe, leading to frequent human interaction [39,40].

This study presents the first description of Caseous Lymphadenitis in a roe deer in Italy.

## 2. Materials and Methods

In March 2022, citizens reported finding a young roe deer carcass in the Appennines of Forlì-Cesena province, Emilia Romagna region (44°03′16.4″ N, 11°50′31.1″ E), to a wildlife rescue association, which arranged for its removal and delivery to the laboratory for necropsy in order to determine the cause of death.

First, the species, sex, age, body weight and body condition were determined. Age was estimated through dental examination [41], and body condition was evaluated based on adipose deposition, muscle mass, and evidence of bone prominences. All necropsy results, including possible traumatic injuries and pathological changes, were recorded. Analyses were conducted according to standard protocols [42] and included gross pathological lesions.

Ticks were collected from various anatomical regions for species identification using a stereomicroscope.

The liver, spleen, lungs, lymph nodes and abscesses were selected for bacteriological examination based on their pathological features. The surfaces of the organs and lesions were sterilized using a Bunsen burner flame before being carved to expose the organ parenchyma.

Material was collected using a sterile inoculation loop or needle and was then directly inoculated on blood agar plates, whereas Hektoen enteric agar was used for the selective growth of Enterobacterales and Pseudomonadales. Both kinds of plates were then incubated at 37 °C for 48 h under both aerobic and anaerobic conditions. Culture media were produced according to ISO 11133:2014 [43] by the laboratories of Istituto Zooprofilattico Sperimentale della Lombardia ed Emilia Romagna (IZSLER, Brescia, Italy). Any isolated microorganism, after Gram staining and the oxidase and catalase test, was identified using MALDI BIOTYPER^®^ (Bruker Daltonics Inc., Billerica, MA, USA), according to the manufacturer’s instructions (Beckman Culter, Inc., S. Kraemer Blvd Brea). Briefly, a single bacterial colony was laid on the carrier and mixed with MALDI BIOTYPER^®^ US IVD Matrix HCCA-portioned. The carrier was then dried until crystallization and loaded into the instrument for ionization with a laser beam. Acceleration at a fixed potential was carried out to separate and sort protonated ions of analytes in the sample based on their mass-to-charge ratio (*m*/*z*). The charged analytes were then detected and quantified using time of flight (TOF) analyzers.

Using API biochemical galleries (bioMérieux, Marcy l’Etoile, France), the nitrate reduction test was carried out.

In accordance with the M45 3rd ed. 2015 CLSI guidelines [44], the antibiotic sensitivity test was performed using the Kirby–Bauer method. Three to five colonies were sterilely collected and diluted in 3 mL of sterile saline solution until a microorganism concentration of 1–2 × 10^8^ CFU, equivalent to the 0.5 McFarland standard, was obtained. The bacterial suspension was then seeded evenly, using sterile buffer, onto two plates of Muller–Hinton Esculin agar, rotating the plates by approximately 60° to cover the entire surface of the medium. The plates were allowed to dry for 3–5 min, and antibiotic discs were then applied with sterile forceps. The tested antibiotics were Amoxicillin; Amoxicillin and Clavulanic Acid; Ampicillin; Cephalothin; Cefoperazone; Chloranphenicol; Enrofloxacin; Gentamicin; Oxytetracycline; Penicillin; Tetracycline; Thiamphenicol; Erythromycin; Bacitracin; Lincomycin; sulfamethoxazole/trimethoprim, and each zone of inhibition was measured with a ruler. Using the published CLSI guidelines, the susceptibility or resistance of the bacterium was determined to each tested drug as susceptible (S), intermediate (I) or resistant (R) based on the interpretation chart.

Portions of lungs and lymph nodes with gross lesions and other organs (liver, spleen, kidney) without macroscopic changes were fixed in 10% buffered formalin for subsequent histological examination.

Parasitological examination for the intestinal (cecum and rectum) content was carried out using 1300 SG flotation solution (composition: sodium nitrate, 540 g; sucrose, 360 g, in 1000 mL of tap water).

## 3. Results

### 3.1. Animal Identification

The carcass examined belonged to an adult female roe deer (*Capreolus capreolus*). The animal weighed 17 kg and its nutritional condition was poor, with deep cachexia, moderate atrophy of the skeletal musculature and particular evidence of bony prominences (ischial and iliac tuberosities, transverse processes of lumbar vertebrae).

### 3.2. Gross Pathology

**Figure 1:**
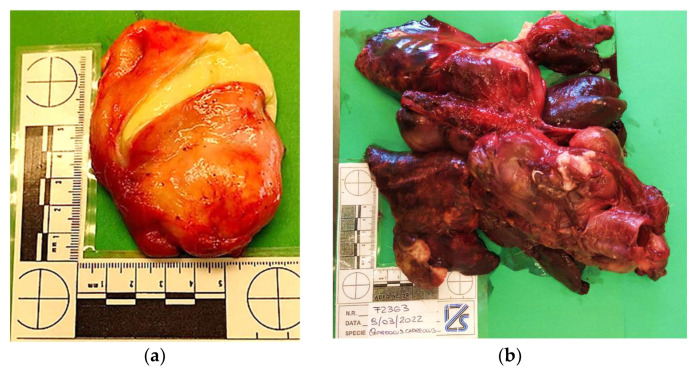
(**a**) Roe deer: purulent lymphadenitis of axillary lymph node. (**b**) Roe deer: severe multifocal abscess purulent pneumonia.

The postmortem condition was adequate and the carcass was fresh. Upon external examination of the carcass, the skin condition was good despite a severe tick infestation, especially in the pectoral and pelvic regions. Palpation led to the identification of generalized lymphadenopathy and subcutaneous lumps in the neck and axillary regions. After flaying, the carving of superficial lymph nodes highlighted purulent to caseous, greenish-yellow and viscous material (Figure 1a), with multifocal and encapsulated features. Thus, purulent lymphadenitis with abscess formation from 3 × 4 cm (neck region) to more than 9 × 7 cm (axillary region) was assessed. Subcutaneous lumps and swellings were also found to be abscesses. Abdominal cavity inspection showed no major alterations, and both the entire gastrointestinal tract and urinary bladder were empty. A pregnancy of at least 3 months was observed after fetus examination, where no macroscopic lesions were identified. In the thoracic cavity, the lungs displayed severe purulent pneumonia with dozens of abscesses of different sizes, ranging from 5 mm to 100 mm (Figure 1b), and large areas of consolidation in the caudal lung lobes. No signs of traumatic injuries were identified.

Bacteriological investigations allowed us to identify the growth of two different types of colonies, one (colony 1) being small, dry and white with defined margins and being surrounded by a beta hemolytic halo, and the other (colony 2) being larger, round, grayish and non-hemolytic, both grown at 37 °C under aerobic conditions in 48–72 h. Colony 1 was shown to be a Gram-positive small rod that is catalase positive and oxidase and nitrate negative, identified as *Corynebacterium pseudotuberculosis;* colony 2 was recognized as *Mannheimia granulomatis*, a Gram-negative coccoid rod that is oxidase positive and catalase negative. Both bacteria were isolated from lung parenchymas and identified by MALDI-TOF. Colony 1 was also obtained from lymph nodes and abscesses.

The antibiotic sensitivity test was performed on *Corynebacterium pseudotuberculosis*, and the results were interpreted after 48 h at 37°± 2 C. The strain was sensitive to all antibiotics tested.

**Figure 2:**
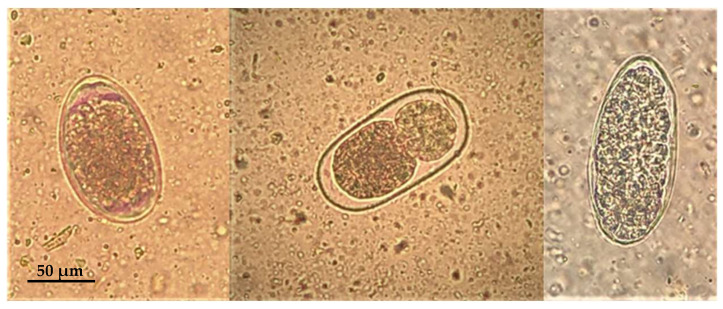
Roe deer: parasite eggs of gastrointestinal strongyles observed in the fecal sample.

Tick examination allowed us to identify three females, one male and one nymph of *Ixodes ricinus* (Linnaeus, 1758) and one female of *Dermacentor marginatus* (Sulzer, 1776). Parasitological examination by flotation revealed a mild infestation by gastrointestinal strongyles (Figure 2).

### 3.3. Microscopic Lesions

**Figure 3:**
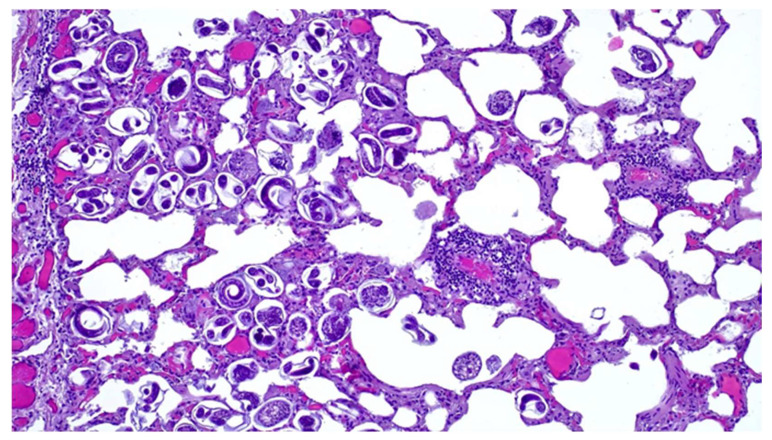
Lung. Histological section. Note the eggs of pulmonary nematodes in various stages of development and first-stage larvae. H&E stain (20×).

Microscopically lung tissue showed a severe parasitic infestation with numerous nematode larvae and eggs present in various stages of development in the bronchioles and the alveolar lumen (Figure 3), consistent with a parasitic bronchopneumonia. A multifocal mild thickening of the alveolar septa, due to the infiltration of macrophages; eosinophils; granulocytes and lymphocytes; hyperemia; the perivascular infiltration of lymphocytes; and peribronchial lymphoid hyperplasia were also observed. Mediastinal lymph nodes showed follicular hyperplasia and the slight infiltration of eosinophil and neutrophil granulocytes, while lymphoid hyperplasia of the splenic white pulp and liver congestion were also observed.

## 4. Discussion

The macroscopic lesions in the roe deer were similar to descriptions of a CLA in sheep, goats and camelids [1,16]. Two main forms have been described in sheep, a superficial or cutaneous form, characterized by the involvement of externally explorable lymph nodes, and a visceral form, associated with abscesses in the internal organs and lymph nodes. Both forms can coexist in the same individual [8], as in this case, in which the animal had suppurative cervical, prescapular and axillary lymphadenitis and simultaneous involvement of the deep airway and loco-regional lymph nodes. Although two different bacteria were isolated in this case as potential agents of abscessual forms, the authors speculated that the disease in the roe deer was CLA itself and therefore attributable to *Corynebacterium pseudotuberculosis*. Supporting this hypothesis is the fact that *Mannheimia granulomatis* was isolated only from lung parenchyma and not from lymph nodes and non-lymph nodes in abscessual lesions, so it is reasonable to consider it, in this case, as an irregular finding without particular pathogenic significance. Some studies in the literature described *Corynebacterium* spp. infections in wildlife caused by *C. pseudotuberculosis*, *C. ulcerans* and novel *C. sylvaticum* with features similar to CLA. For example, a survey in South Africa identified 25 antelopes of different species with a suppurative systemic infection determined by *C. pseudotuberculosis* [45]. In a study by Muller (2011) [45], affected animals with lesions mostly located in the lung and the mediastinal lymph node had similar characteristics to those presented in this study: however, a co-infection with many different non-tuberculosis *Mycobacteria* was observed. Instead, no *Mycobacteria* were detected in the present study.

Wild *Caprinae* species have been reported to be infected with CLA; for example, a captive population of Iberian Ibex in Spain showed a 19% positivity rate for *C. pseudotuberculosis* antibodies [26]. An Italian case study of CLA in Alpine Ibex with purulent collections in various lymph node groups (in particular, subcutaneous and mediastinal) [27] was followed by a comprehensive survey of 98 alpine chamois, which thoroughly described the disease in many different aspects [28]. Domenis et al. (2018) [28] describe four different gross lesion distribution patterns, observed individually or variably combined in the same animal (cutaneous/external, abdominal visceral, thoracic visceral and generalized visceral). The different anatomopathological pictures observable would reflect the different routes of entry of the microorganism (e.g., traumatic skin lesions for superficial forms, the aerogenic route for visceral thoracic forms, the genital or mammary route for abdominal forms and dissemination by lymphatic and hematogenous routes for generalized forms). Based on the number of cases and surveys for *Caprinae*, it appears that wild *Caprinae* have a higher prevalence of *C. pseudotuberculosis* than cervids. It could be speculated that wild *Caprinae* (e.g., Chamois and Alpine Ibex) may have a higher likelihood of transmission between individuals, as they tend to live in larger groups than roe deer [41,46].

Only a few studies on CLA or *Corynebacterium* spp. infections in cervids have been described in the literature, and they are listed in Table 1. Briefly, a local infection by *C. ulcerans* has been described in a German case study of a roe deer [5], which was subsequently referred to as *C. sylvaticum* [22]. *C. pseudotuberculosis* has been described in a captive elk in the USA with subcutaneous abscesses and weight loss, ultimately leading to death [30]. CLA has been described in wild cervids in the form of local abscesses [29,32] or systemic pathology [31,33]. One study described CLA caused by *C. pseudotuberculosis* in a single roe deer, during a fifty-year surveillance on roe deer in Switzerland [34]. As can be observed in Table 1, the studies reported in the present review all refer to a few cases or even single animals.

Two subtypes of *C. pseudotuberculosis* are known, one capable of reducing nitrates to nitrites and classified as biovar ovis, and the other lacking this biochemical characteristic and referred to as biovar equi. The former is mainly isolated from domestic and wild small ruminants, while the latter is isolated from horses and cattle. The strain isolated in this case was classified as biovar ovis, similar to other reports on cervids [32].

The lesions were mostly associated with single to diffuse abscesses in different regions. This feature may be related to the most common entry route of *C. pseudotuberculosis* (i.e., skin and mucous membranes) caused by fights, predation or any other minor wounds. In the current study, the abscessed lymph nodes were distributed in different body regions, but only the respiratory system was involved. Therefore, the entry route could have been one of the abscesses found in the thoracic or cervical regions, or directly through the respiratory system. No genital or abdominal lesions were found, and the fetus was in good condition. The generalization of the lymphadenopathy, as well as the characteristics of the abscess content of the lesions, which is strongly creamy, liquefied and lacks the typical “onion ring” structure, can be reasonably explained by the particular aggressiveness of the strain involved and/or the inadequacy of the immune system to counteract the spread of the microorganism, probably also related to the state of pregnancy.

The strain isolated in this case did not show antibiotic resistance to any of the antibiotics tested, a finding that is particularly important with respect to beta-lactams, the class of molecules most commonly used in the treatment of pseudotuberculosis in domestic animals.

Bacterial pulmonary pathology associated with *C. pseudotuberculosis* is exacerbated by verminous bronchopneumonia related to the presence of high numbers of nematode eggs and larvae, which is particularly severe in the caudal lobes. Several studies have reported a prevalence of *Dictyocaulus* spp. in the lungs of roe deer in fairly similar climatic areas [47,48,49]. Although the pathogenic significance of nematode infestation in wild ruminants is still controversial, some authors have hypothesized that susceptibility to infestation is closely linked to the poor condition of the animal [50], so we could assume that the severity of parasitic pneumonia is related to, or perhaps a contributing cause of, the severely compromised health status of the animal, creating conditions for bacterial dissemination.

## 5. Conclusions

To the best of the authors’ knowledge, this is the first described case of caseous lymphadenitis in a roe deer in Italy. Unfortunately, very little is known about the true epidemiology of this infection in wildlife worldwide, as most of the carcasses that are reported and recovered for further diagnostic investigation belong to animals that are most often found by the roadside and are thus victims of road accidents. However, the diagnostic activity conducted as part of the passive surveillance carried out routinely on wildlife by the Experimental Zooprophylactic Institutes for their area of responsibility is an essential tool for acquiring information regarding the presence, circulation, even in different animal populations, and geographic spread of certain pathogens that may represent a health and economic problem in domestic species as well as zoonotic, especially for professional categories in contact with affected animals. Infectious diseases represent a major threat to wildlife, as the shedding of pathogens from domestic animals into the environment and thus to wildlife is frequently described. The possible reservoir-like role of roe deer for *C. pseudotuberculosis* is not yet fully understood but may have implications for wild species, conservation and the wildlife/domestic animal/human interface, especially in areas where infected flocks are common. Surveys on *Corynebacterium* spp. in cervids can be a useful tool to better understand the disease in wildlife and to assess the epidemiological status of a particular area.

## Figures and Tables

**Table 1:** *Corynebacterium* spp. infections in wild cervids: published studies.

Study	Cervid Species	Animals Involved (N°)	Corynebacterium Species and Identification Method	Organs/Regions Affected
Staber 1973 [29]	White-tailed Deer (*Odocoileus* *virginianus*)	1	*C. pseudotuberculosis* Culture	Thoracic cavity
Kelly 2012 [30]	Rocky Mountain Elk (*Cervus canadensis* *nelsoni*)	2	*C. pseudotuberculosis* Culture	Head abscess
Matos 2014 [31]	Red Deer (*Cervus elaphus*)	5	*C. pseudotuberculosis* PCR	Mesenteric lymph nodes
Hussain 2017 [33]	Spotted Deer (*Axis axis*)	3	*C. pseudotuberculosis* PCR	Not defined
Morales 2017 [32]	Patagonian Huemul (*Hippocamelus* *bisulcus*)	2	*C. pseudotuberculosis* culture	Cutaneous abscess
Pewsner 2017 [34]	Roe deer	1	*C. pseudotuberculosis* culture	Systemic
Dangel 2020 [22]	Roe deer	1	*C. sylvaticum*	Cutaneous abscess

## Data Availability

Data are contained within the article.
